# Effect of Rhythmic Auditory Cueing on Aging Gait: A Systematic Review and Meta-Analysis

**DOI:** 10.14336/AD.2017.1031

**Published:** 2018-10-01

**Authors:** Shashank Ghai, Ishan Ghai, Alfred O. Effenberg

**Affiliations:** ^1^Institute for Sports Science, Leibniz University Hannover, Germany; ^2^School of Life Sciences, Jacobs University Bremen, Germany

**Keywords:** cueing, stability, rehabilitation, cognitive-motor interference, balance, entrainment, dual task

## Abstract

Rhythmic auditory cueing has been widely used in gait rehabilitation over the past decade. The entrainment effect has been suggested to introduce neurophysiological changes, alleviate auditory-motor coupling and reduce cognitive-motor interferences. However, a consensus as to its influence over aging gait is still warranted. A systematic review and meta-analysis was carried out to analyze the effects of rhythmic auditory cueing on spatiotemporal gait parameters among healthy young and elderly participants. This systematic identification of published literature was performed according to PRISMA guidelines, from inception until May 2017, on online databases: Web of science, PEDro, EBSCO, MEDLINE, Cochrane, EMBASE, and PROQUEST. Studies were critically appraised using PEDro scale. Of 2789 records, 34 studies, involving 854 (499 young/355 elderly) participants met our inclusion criteria. The meta-analysis revealed enhancements in spatiotemporal parameters of gait i.e. gait velocity (Hedge’s g: 0.85), stride length (0.61), and cadence (1.1), amongst both age groups. This review, for the first time, evaluates the effects of auditory entrainment on aging gait and discusses its implications under higher and lower information processing constraints. Clinical implications are discussed with respect to applications of auditory entrainment in rehabilitation settings.

Higher prevalence to fall with aging is a matter of concern for medical practitioners [1-3]. According to WHO, every year approximately 37 million people are seriously injured, and further 424,000 people perish from falls globally [4]. Degenerative changes in cardiovascular [5], sensorimotor (somatosensory, vestibular), and neuromuscular (cortical, extra-pyramidal, cerebellum) domains are suggested to be the main reasons often leading to falls [6-8]. Moreover, medications, depression, and anxiety are additional precipitators [9-11]. Falls impact quality of life [12, 13], and inflict heavy costs at both individual, economic levels [14, 15].

Studies suggest that highest incidences for falls occur during locomotion [16-18]. In fact, aging has been associated with modifications in spatiotemporal [19], electromyographic [20], and kinematic [21], gait parameters, which in-turn are important predictors for fall. For instance, clinical characteristics for reductions in gait velocity, stride length, cadence, single limb support phase, and enhancements in stride time, double limb support phase, gait variability [22, 23], have been well documented (see also Jahn, et al. [6]). The kinematic analysis also suggests a reduction in angular impulse, torque at ankle, knee, and hip joint with aging gait [24]. Together, these factors aggravate static and dynamic instability and increase predisposition to fall. Likewise, degenerative changes observed in psychological domain in elderly might also contribute in modifying stability [25, 26], and cognitive processing [27, 28]. Reelick, et al. [23], for instance, suggested a reduction in self-confidence with aging, and history of falls often leading to a peculiar “fear of falling” [29, 30]. Furthermore, this “fear” has been reported to additionally modify the stability during static, and dynamic postures [9, 31, 32]. Giladi, et al. [32], referred such modified gait pattern as a “cautious or fearful gait” [23]. Although these modifications are aimed to enhance stability during locomotion, they, in turn, develop a stiff, slow and unsteady gait pattern [33]. Moreover, this “fear of falling” or “cautious gait” might promote “internal” attentional focus [34], explicit motor control [25], and can eventually alleviate cognitive-motor interferences [35] (see also Young and Mark Williams [33]). Masters and Maxwell [27] suggested that such an attempt to consciously monitor or control an autonomic movement, such as posture, or gait might adversely affect its performance. Also, such higher information processing constraints have demonstrated detrimental effects on proprioceptive perceptions [36-38], which are integral for autonomic stability [36]. In addition, literature suggests that younger population groups, on the contrary, have a more resilient and stable psycho-physiological stature [35, 39]. However, falls are not uncommon [10]. Possibly, environmental [10], and lifestyle factors might play a considerable role [40]. Schabrun, van den Hoorn, Moorcroft, Greenland and Hodges [41] reported texting and reading while walking (common among youngsters) to adversely impact gait stability [42], by increasing cognitive-motor interferences [43]. Consequently, such higher attentional constraints predisposing to falls might possess serious life-threatening consequences under “high-stress” environments [8, 44], for both younger and elderly age groups.

Several strategies have been suggested in literature to curb these psycho-physiological deficits, such as pharmacotherapy (Methylphenidate) [5], virtual-reality [45], biofeedback [46], physical/occupational therapy [47], physical exercise [48], dance [49], treadmill [50], external sensory cueing [51, 52], martial arts [53, 54], dual-task training [5, 36], and more [55]. Amongst these, external sensory entrainment in rehabilitation is an emerging yet under-evaluated area of interest [56]. For instance, external auditory cueing can enhance motor performance in patients with sensorimotor deficits [57], even better vis-a-vis tactile and visual entrainment [56-59]. Possibly, due to lower rhythm perceptional thresholds for auditory cortex [56, 60, 61], rich neural connectivity [52, 62, 63], and better temporal precision [52, 62, 63]. Moreover, published literature suggests beneficial effects auditory entrainment during gait amongst patients affected from traumatic neurological injuries [64], multiple sclerosis [65], stroke [66], parkinsonism [57], and even healthy young and elderly participants [67, 68]. The auditory entrainment might supplement sensory deficits present in fall prone individuals [69], and aid in performance by mediating multifactorial neurophysiological changes [52, 70], enhancing auditory imagery [71-74], reducing variability in musculoskeletal activation [75], and possibly cognitive-motor interference [67, 76].

Additionally, rhythmic auditory entrainment is cheap [77], viable [78], easy to follow and has shown enhancements even during unsupervised home-based training programs [79, 80]. This intervention can be a useful rehabilitation tool in middle and lower income countries, where poor healthcare services [81], might precipitate to majority of the fall related deaths [4]. Thereby, strongly warranting the need for such economical, and efficient rehabilitation techniques.

High-quality systematic reviews and meta-analyses have been carried out to evaluate the beneficial effects of rhythmic auditory cueing on gait in patients affected from neurological conditions, such as stroke, and parkinsonism [57, 58, 66]. However, to the best of our knowledge, no review to date has analyzed the effects of rhythmic auditory cueing on aging gait. Therefore, we attempted to develop a state of the art knowledge for the use of rhythmic auditory cueing in gait rehabilitation across healthy population groups. The main aim of this review is to understand the effects of auditory entrainment on spatiotemporal, variability parameters for gait among young, and elderly age groups. The review also discusses possible applications of auditory entrainment in rehabilitation and activities for daily living.

## METHODS

This review was conducted according to the guidelines outlined in Preferred Reporting Items for Systematic Reviews and Meta-analysis: The PRISMA statement [82].

### Data sources and search strategy

Academic databases such as Web of science, PEDro, EBSCO, MEDLINE, Cochrane, EMBASE and PROQUEST were searched from inception until July 2017. A sample search strategy has been provided in (Supplementary Table 1).

### Data extraction

Upon selection for review, the following data were extracted from each article; author, date of publication, selection criteria, sample size, sample description (gender, age, health status), disease duration, intervention, characteristics of auditory feedback, dual-task, outcome measures, results, and conclusions. The data were then summarized and tabulated (Table 1).

The inclusion criteria for the studies was (i) Performed studies were either randomized controlled trials, cluster randomized controlled trials or controlled clinical trials; (ii) Studies reporting reliable and valid spatiotemporal gait parameters (iii) Studies reporting dynamic aspects of gait stability (iv) Studies qualified PEDro methodological quality scale (≥4 score); (v) Experiments conducted on human participants; (vi) Published in a peer-reviewed academic journal; (vii) Articles published in English and German languages.

### Quality & risk of bias assessment

The quality of the studies was assessed using the PEDro methodological quality scale [83]. The scale consists of 11 items addressing external validity, internal validity, and interpretability and can detect potential bias with fair to good reliability [84], and validity [83]. A blinded rating of the methodological quality of the studies was carried out by the primary reviewer. Ambiguous issues were discussed with second (IG), third (AOE) reviewer and consensus was reached. Included studies were rated, and interpreted according to scoring of 9-10, 6-8 and 4-5 considered of “excellent”, “good” and “fair” quality [85], respectively. Inadequate randomization, non-blinding of assessors, no intention to treat analysis and no measurement of compliance were considered as major threats to biasing [86].

**Figure 1.**
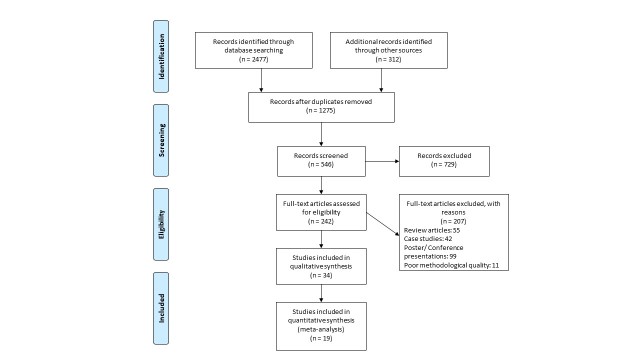
PRISMA flow chart for the inclusion of studies.

### Data Analysis

This systematic review included a meta-analysis approach [87]. The presence and lack of heterogeneity asserted the use of either random or fixed effect meta-analysis [88], respectively. A narrative synthesis of the findings structured around the intervention, population characteristics; methodological quality (Table 1) and the type of outcome are provided. Likewise, summaries of intervention effects for each study are also provided in a tabular form (Table 1). A meta-analysis was conducted between pooled studies using CMA (Comprehensive meta-analysis V 2.0, USA). Heterogeneity between the studies was assessed using I^2^ statistics. The data in this review was systematically distributed and for each available variable pooled, dichotomous data was analyzed and forest plots with 95% confidence intervals are plotted. The weighted effect sizes are reported as Hedge’s g [89]. Thresholds for interpretation of effect sizes were as follows; a standard mean effect size of 0 means no change, negative effect size means a negative change, mean effect size of 0.2 considered a *small* effect, 0.5 a *medium* effect and 0.8 a *large* effect [90]. Interpretation of heterogeneity via I^2^ statistics was as; 0-0%, 25%, 75% as negligible, moderate and substantial heterogeneity, respectively. Meta-analysis reports including heterogeneity among studies were evaluated to determine the reason of heterogeneity, and the included studies were then pooled separately and analyzed again. The alpha level was set at 95%.

## RESULTS

### Characteristics of included studies

Our initial search yielded a total of 2789 studies, which on implementing our inclusion/exclusion criteria, were reduced to thirty-four (Fig. 1). Data from the included studies have been summarized in (Table 1). Of the thirty-four included studies, one was randomized controlled trial, and thirty-three were controlled clinical trials.

**Table 1:** Studies analyzing the effects of rhythmic auditory cueing on gait.

Author	Sample description, age: (M ± S.D years)	PEDro score	Assessment tools	Research design	Auditory feedback elements	Conclusion
Dotov, et al. [100]	7F, 12M (60)	6	Coefficient of variation of inter-stride interval, cadence, gait velocity, stride length, DFA of short-long term series of inter-response-interval correlations, circular statistics for synchronization of footfall & beat	Pre-test, gait performance with/without RAC (no variability, biological variability, non-biological variability; randomized), post-test	RAC with no variability, biological variability & non-biological variability at +10% of preferred cadence Magnitude of biological & non-biological variability: 2% of inter-beat-interval Metronome sequence: triangle timbre Musical excerpts Amplitude modulated noise: Modulated on musical excerpt with drum ensemble, discarding tonal information	Significant enhancement in coefficient of variation for inter-stride interval after RAC in all conditions. Significant effect of RAC that was amplitude modulated for biological variability as compared to IC on short-long term correlation for term series of inter-response-interval correlations. Enhanced synchronization, cadence but reduced short-long term correlation for term series of inter-response-interval correlations during metronome based IC as compared to feedback with amplitude modulated for biological variability.
Maculewicz, et al. [141]	5F, 15M (24.4±3.2)	4	Mean square error for the asynchrony between target & performed measure & trend of tempo change obtained from slope of line fitted to measured tempo, questionnaire	Gait performance with/without real-time auditory feedback (adaptive), RAC (constant) &/or haptic feedback, with instructions to perform gait at preferred cadence or the tempo of the sound	Real-time auditory feedback (adaptive), RAC (constant) by sine, wood & gravel sounds	Significantly enhanced step wise interaction with real time auditory feedback with (sinusoid >wood>gravel). Significant reduction in asynchrony with audio-haptic feedback & real-time auditory feedback as compared to no feedback. Significant enhancement in comfort for perceiving haptic & audio-haptic feedback as compared to haptic only or no feedback in self-reported questionnaire.
Schreiber, et al. [97]	5F, 12M (37.4±15.7)	4	Cadence, gait speed, rhythmicity, stance time, double support time, gait symmetry, step length, stride length, step width, EMG activity of (tibialis anterior, soleus, gastrocnemius medialis, vastus medialis, rectus femoris, semitendinosus, gluteus medius & gluteus maximus), kinematics for pelvis, hips, knees & ankle joint (sagittal, frontal, transverse plane)	Gait performance with/without RAC cueing at preferred, reduced cadence (instructions & cueing randomized)	RAC at preferred & reduced cadence	Significantly reduced gait speed with RAC at preferred cadence as compared to preferred speed gait without cueing. No effect of RAC on cadence, rhythmicity, stance time, double support time, gait symmetry for RAC at preferred or reduced cadence as compared to no cueing. Significantly reduced step width with RAC at reduced cadence as compared to reduced speed gait without cueing. Significantly enhanced step length with RAC at reduced cadence as compared to reduced speed gait without cueing. Significant differences for ankle dorsiflexion, hip flexion & hip abduction of the gait cycle with RAC at reduced cadence as compared to reduced speed gait without cueing.
Hamacher, et al. [104]	Young: 8F, 12M (24.9±4.1) Old: 11F, 9M (67.4±5.3)	5	Stride length, minimum foot clearance, stride time, stride to stride analysis (mean & coefficient of variation)	Gait performance with/without dual-task (arithmetic subtraction in 3’s task) &/RAC (randomized)	RAC at preferred cadence	Significant enhancement in stride length, stride time with RAC (with/without dual-task) in both younger & older adults. Significantly enhanced coefficient of variation of stride time in older participants under dual-task condition & with RAC Enhancement in coefficient of variation of stride to stride in older participants under dual-task condition & with RAC
Terrier [96]	22F, 14M (33±10)	4	DFA of coefficient of variability for stride time, stride length, stride speed, stride length, stride speed & stride time	Gait performance on treadmill with/without visual (stepping stones), RAC	RAC at preferred cadence	Significant reduction in stride time & stride speed with RAC as compared to no cueing. No effect on coefficient of variation for stride length, stride time & stride speed (mean & coefficient of variation) with RAC
Roerdink, et al. [162]	5F, 7M (28±6)	5	Stride-to-stride DFA for persistence of stride time, stride length, stride speed & anterior-posterior center of pressure sway	Treadmill gait performed with/without RAC with isochronous metronome & non-isochronous metronome containing inter-beat interval sequences with distinct scaling exponents (randomized)	RAC with (IC) containing equidistant inter-beat interval & 4 (non-isochronous) metronome containing inter-beat interval sequences with distinct scaling exponents Frequency: 600Hz RAC with mean inter-beat intervals being equal to mean stride time of preferred cadence.	Significant effect of IC cueing for changing the stride-to-stride fluctuations of stride length & stride time to anti-persistent & vice versa for the non-IC. Significant effect of isochronous & non-isochronous metronome cueing for changing the stride-to-stride fluctuations of stride speed to anti-persistent for both the cueing.
Wright, Spurgeon and Elliott [163]	8F, 2M (20-33)	5	Mean asynchrony, step time variability & mean percentage step correction	Gait performance with/without RAC &/or visual cueing	RAC, 500 ms (cue duration 30 ms), 800Hz	Significant enhancement in & mean percentage step correction with audio & audio-visual cueing as compared to only visual cueing Significant reduction in mean synchrony of step with RAC with audio-visual cueing as compared to only audio or visual cueing. Significant reduction in step time variability with audio & audio-visual cueing as compared to only visual cueing
Young, et al. [138]	6F, 4M (63.9±4) II: same as I III: same as I	5	I: Mean step length, % change stride length, mean step duration, % change in variability of stride length, duration II: same as I III: same as I	I: Gait performance with/without verbal instruction, verbal instruction-metronome cueing, stepping sound, stepping sound-verbal instructions, for small and wide stride length (randomized) II: Gait performance with/without stepping sound, verbal instruction-stepping sound feedback, synthesized gravel sound, synthesized gravel sound-verbal instructions, for small and wide stride length (randomized) III: Gait performance with/without motor imagery, motor imagery-stepping sound feedback, synthesized gravel sound, synthesized gravel sound-motor imagery, for small and wide stride length (randomized)	I: RAC (Ct: 550-649ms, Exp: 600-700ms), foot step feedback on gravel (500, 600, 700ms) II: RAC (Ct: 550-649ms, Exp: 600-700ms), foot step feedback on gravel (500, 600, 700ms), synthesized gravel step sound corresponding to plantar force (developed by using ground reaction forces vector to modulate both intensity envelop, and central frequency of bandpass filter applied to stochastic noise impulse signal) III: same as II	Significant enhancement in stride length for healthy Ct in all cueing conditions. No effect of auditory cueing or instructions on mean step duration. Significant reduction in stride length variability with synthesized feedback as compared to footstep feedback-verbal instruction, synthesized feedback-verbal instructions. Significant reduction in stride length variability with stepping, synthesized feedback, stepping-verbal instructions. Significant enhancements in stride length with rhythmic auditory cueing (synthesized) and motor imagery together. No effect on stride duration parameters.
Leow, et al. [105]	24F, 19M (18-20)	5	Stride velocity, step length, step time, stride width, double support, & coefficient of variability for stride length	Gait performance with/without rhythmic music, RAC (low/high groove) at 0% & +22.5% of preferred cadence	RAC (low/high groove music) at 0% & +22.5% of preferred cadence (50ms 1kHz sine tones)	Significant enhancement in stride velocity with rhythmic music cueing (high groove) & metronome at +22.5% of preferred cadence as compared to no cueing. Significant reduction in double support with metronome cueing at 0% & +25% of preferred cadence as compared to no cueing. Significant reduction in step length with high groove music at +25% of preferred cadence. Significant reduction in step time in low (0% also), high groove music cueing & RAC at +25% of preferred cadence cueing as compared to no cueing. Significant enhancement in coefficient of variability for stride length with low & high groove RAC at 0% & +25% of preferred cadence.
Sejdić, et al. [164]	8F, 7M (23.9±4.7)	5	Gait speed, mean stride interval, stride interval variability, stride interval dynamics, dynamic stability of gait in anterior-posterior, vertical & medio-lateral dimension (short: between 0^th^ & 1^st^ stride & long: between 4^th^ & 10^th^ stride, term Lyapunov exponent)	Gait performance with rhythmic auditory, visual & haptic cueing (randomly spate or together) at preferred cadence during 2 sessions	RAC at preferred cadence	Significantly reduced stride interval variability with RAC (alone & combined with visual & haptic cueing) as compared to no cueing condition. Significantly reduced stride interval dynamics (long term Lyapunov exponent) with RAC (alone & combined with visual & haptic cueing) as compared to no cueing condition. Significant enhancement in dynamic stability of gait with RAC (alone & combined with visual & haptic cueing) as compared to no cueing condition.
Terrier and Dériaz [165]	10F, 10M (36±11)	4	DFA on time series of stride time, stride length & stride speed Short & long-term local dynamic stability in anterior-posterior & medial-lateral direction	Gait performance on treadmill at slow (0.7 times preferred cadence), fast (1.3 times preferred cadence) & at preferred cadence with/without RAC (randomly)	RAC at slow (0.7 times slower than preferred cadence), fast (1.3 times faster than preferred cadence) cadence	Significant enhancement in long term local dynamic stability with RAC Significant reduction of stride time & stride length variability at slow speed with RAC No effect on short term local dynamic stability with RAC
Roerdink, et al. [166]	10F, 10M (63.2±3.6)	5	Cadence, mean relative timing between footfalls & auditory stimuli, variability of mean relative timing (by circular statistics)	Participants performed gait at preferred cadence followed by 7 random trials with adjusted RAC i.e. 77.5%, 85%, 92.5%, 100%, 107.5%, 115% or 122.5%	Auditory input from drum RAC at 77.5%, 85%, 92.5%, 100%, 107.5%, 115% or 122.5% of preferred cadence Different pitch to pace for RAC i.e. for step left: 440Hz, right: 1000Hz	Significant effect of RAC on cadence, mean relative timing & variability of mean relative timing between footfalls & auditory inputs. Significantly fewer steps required to reach synchronization
Lohnes and Earhart [67]	Young: 7F, 4M (24±0.8) Old: 7F, 4M (70.8±10.4)	5	Gait velocity, cadence & stride length	Patients performed gait with/without RAC at -10%, +10% of preferred cadence or with additional cueing strategy “think about larger strides” with/without -10% & +10% of auditory inputs tone, with/without dual-task “word generation task”	RAC at ±10% of preferred cadence.	Significant effect on gait velocity stride length, cadence for both groups with ±10% of RAC under both single and dual-task conditions. Larger effects noted in young participants as compared to older counterparts. Verbal instructions had no influence on cadence among both groups under both single and dual-task conditions.
Trombetti, et al. [167]	Exp: 64F, 2M (75±8) Ct: 65F, 3M (76±6)	8	Gait velocity, stride length, cadence, double, single support phase, stride time/length variability, TUG test, trunk angular displacement, Tinetti tests & assessment of falls	Exp: Pre-test, gait & exercise training with auditory input performed for 1-hour session/week for 12 months, 6-month test, post-test, with/without dual-task (counting backward aloud task) Ct: started 6-month delayed intervention, with/without dual-task (counting backward aloud task)	RAC as piano music	Single task: Significant enhancement in gait velocity, stride length & stride time variability for the Exp as compared to Ct. Dual-task: Significant enhancement in stride length, decrease in stride length variability in Exp as compared to Ct Significant enhancement in 1 legged stance, Tinetti tests, TUG & decreased mediolateral angular velocity. Significantly reduced incidences of falls in Exp as compared to Ct.
Wittwer, et al. [136]	12F, 7M (79±7.8)	4	Swing time, stride time, velocity, stride length, double support %, stride width, stride length & time variability	Participants performed gait with/without auditory feedback “randomly” i.e. music or RAC	Music or metronome or RAC at participants preferred cadence	Significant enhancement in velocity, stride length with music as compared to no sound. Significant reduction in stride time, double limb support & enhancement in cadence with both music & RAC input, as compared to no auditory input. No effect on mean step width, mean temporal or spatial gat variability.
Yu, et al. [93]	13F (21.8±0.4)	5	Stride length, cadence & gait speed	Gait performance with/without RAC at 0% & ±10% of preferred cadence	RAC at 0% & ±10% of preferred cadence	Significant enhancement in stride length, cadence & gait speed with +10% RAC as compared to all conditions. Significant reduction in cadence & gait speed with -10% of RAC as compared to 0% & no cueing.
Almeida, et al. [92]	Exp I: 9 (42.7±6.6) Exp II: 10 (42.4±4.5) Ct: 9 (41.7±5)	4	Gait speed, heart rate, maximal oxygen consumption, rating of perceived exertion	Gait performance with/without (Ct) RAC at 90 bpm (Exp II) & 140 bpm (Exp I) for 30 minutes with re-tests at every 5-minute interval	RAC at 90 & 140 bpm	Significant enhancement in gait performance in Exp I as compared to Exp II & Ct. No effect on heart rate & maximal oxygen consumption in Exp or Ct.
Hunt, McGrath and Stergiou [168]	4F, 6M (28.1±5.3)	4	Stride time, sample entropy of stride time interval for individualized fractal RAC, DFA for auditory signals scaling exponent & stride time scaling exponent	Gait performance with/without individualized fractal RAC for white, pink & brown noise (randomized)	Individualized fractal RAC (embedding white, pink & brown noise variables into inter-beat interval of music) Inter-beat interval: stretched or compressed based on dynamics of pink, white or brown noise time series Amplitude: standard deviation of inter-beat intervals matched standard deviation of step time Tempo: at preferred cadence	Significant effect of RAC on sample entropy of stride interval time series (brown>pink>white>no sound) Significant enhancement of fractal scaling exponent with auditory feedback of stride interval time series (brown>pink>white>no sound)
Marmelat, et al. [169]	7F (28±6)	5	DFA of inter slide interval variability, inter-beat interval variability & asynchrony with metronome between two successive right heel strikes	Gait performed on treadmill with/without RAC with either IC or fractal feedback	RAC with either IC or fractal feedback Inter-beat intervals contained fractal Gaussian noise with corresponding scaling exponent (600 Hz)	Significant effects of pacing rhythmic metronome feedback on global exponents of inter-beat & slide intervals (persistent correlations) No effect on inter slide interval, asynchrony with RAC Participants anticipated the metronome & adapted with pacing stimuli No significant correlations between inter-beat intervals & inter-slide intervals (increased correlation with increased variability)
	5F, 7M (28±6)	5	DFA of inter slide interval variability, inter-beat interval variability & asynchrony with metronome between two successive right heel strikes	Gait performed on treadmill with/without RAC with either IC or fractal feedback	RAC with non-IC (different scaling exponents)	Significant effects of pacing rhythmic metronome feedback on global exponents of inter-slide intervals (anti-persistent correlations) No significant correlations between inter-beat intervals & interslide intervals (increased correlation with increased variability)
Franěk, et al. [68]	30F, 42M (20.2±1.2)	4	Gait speed, synchronization (inter step times)	Gait performed with/without rhythmic music feedback at 114, 124, 133 bpm	RAC at 114, 124, 133 bpm	Significant enhancement in gait speed with faster tempo music feedback as compared to slower tempo RAC & no feedback. No effect on synchronization with rhythmic music feedback.
	60F, 61M (20.6±1.5)	4	Gait speed, synchronization (inter step times)	Gait performed with/without] RAC (music motivational/non-motivational)	RAC (music motivational: 131-200 bpm, non-motivational: 52-96 bpm)	Significant enhancement in gait speed with motivational rhythmic music feedback as compared to non-motivational RAC & no feedback.
Leman, et al. [142]	11F, 7M (22-51)	4	Gait speed, gait tempo, synchronization of steps to tempo	Gait performance with 52 rhythmic music excerpts (activating & relaxing)	RAC (relaxing or activating effects) at 130 beats per minute, short fade in of 50 ms & fade out of 100 ms applied to each musical excerpt RAC superimposed at position 1, 12, 23, 34, 45, & 58	Significant effect of activating (increased gait speed), relaxing (reduced gait speed) in gait speed with RAC with same tempo. Significant enhancement in synchronization of steps with RAC
Peper, et al. [170]	Young: 4F, 8M (22-28) Old: 5F, 7M (55-69)	5	Mean reaction time, gait speed, step length, step width	Gait performed with/without RAC & visual feedback (stepping stones), dual-task (probe reaction task generating vibrating stimuli)	RAC Left (440Hz), right (1000Hz) Temporal shift of ±1/6^th^ of interval between consecutive ipsilateral beeps, causing ±60º phase delay/advance	Significantly enhanced step length & step width RAC No effect on gait speed in young & older adults with RAC Significantly enhanced reaction times with RAC as compared to no cueing. Significantly reduced reaction time with RAC as compared to visual cueing.
Bank, Roerdink and Peper [171]	10F, 10 M (63.2±3.6)	5	Mean normalized step time, step length, relative phase shift between gait & cues	Gait performance with RAC ±22.5% (introduced in steps of ±7.5% randomly) of preferred cadence &/or stepping stone visual feedback	RAC at ±22.5% of preferred cadence Temporal shift of ±1/6^th^ of interval between consecutive ipsilateral beeps, causing ±60º phase delay/advance	Significant effect of phase delay on increasing/decreasing step length, step time with auditory & visual feedback. However visual cueing > RAC Significantly enhanced phase shift from auditory to visual cueing condition. Significant reduction in coordination of RAC with gait as compared to visual cueing
Wellner, et al. [91]	17 (28±8)	4	Obstacle hit %, average obstacle clearance & individually chosen gait speed	Gait performance on robot assisted device with/without Rhythmic auditory feedback (distance to obstacle &/or foot clearance feedback)	Rhythmic real-time feedback for distance to obstacle & foot clearance Obstacle distance: Rhythm (repeating sound with shorter pause interval as distance decreases), continuous/discrete pitch (continuous sound with higher pitch as distance increases/decreases), dynamics (increase in volume as distance decreases) Absolute foot clearance: harmony (dissonant/consonant chords below/above obstacle), pitch with 2 & 3 levels, noise (Gaussian noise below, no sound above obstacle)	Significantly enhanced self-chosen gait speed with auditory feedback as compared to only visual feedback. Significant enhancement in gait speed with rhythmic feedback for distance to obstacle &/or foot clearance as compared to no feedback
Arias and Cudeiro [102]	6F, 5M (65.7±7.6)	5	Cadence, gait velocity, step amplitude, coefficient of variation for step amplitude & stride time	Patients performed gait with/without rhythmic cueing from auditory, visual & audio-visual condition, with frequency ranging from 70-110% increment/decrement at ±10% of preferred cadence	RAC with wave frequency of 4625 Hz delivered at frequency ranging from 70-110% increment/decrement at ±10% of preferred cadence	Significant enhancement in cadence, step amplitude in Ct with RAC No effects on gait velocity, coefficient of variability for stride time & stride amplitude.
Baker, et al. [172]	7F, 5M (71.5±2.5)	7	Gait speed, coefficient of velocity for (step time, double limb support time)	Pre-test, functional gait performance with/without RAC -10% of preferred cadence, attentional cue instructions "try to take big steps", together "take a big step with the beat", & with/without a dual-task (a tray with 2 cups of water on top), post-test	RAC at -10% of preferred cadence	Significant effect of RAC back and verbal instructions on enhancing stride length, gait velocity. Significantly reduced cadence with RAC and verbal instructions. Reduced gait speed, cadence with -10% RAC No effect on stride length.
Hausdorff, et al. [117]	14F, 12M (64.6±6.8)	5	Stride time, gait speed, stride length, swing time, stride time variability & swing time variability	Pre-test, gait performance with/without RAC at preferred cadence, +10%, Post-test 2 & 15 min short term retention test	RAC at 0% & +10% of preferred cadence	Significant enhancement in gait speed with +10% RAC Significant reduction in stride time with +10% RAC No effect on stride length, swing time, stride time variability, swing time variability with RAC
Willems, et al. [103]	9 (68.1±7.3)	5	Steps (number, time, height, width, length), step length, step width, step duration, coefficient of variation of step duration	Gait performance while turning with/without RAC	RAC at preferred cadence	Enhancement in step length. No effects on steps (number, time, height, width), step length, step width, step duration, coefficient of variation of step duration with RAC
Baram and Miller [99]	6F, 5M (25.4±1.9)	4	Gait speed, stride length, 10 meters walking test	Pre-test, followed by rhythmic auditory feedback & 10 min follow-up short term residual performance test	Rhythmic auditory feedback generated with gait step in real-time	No effects on stride length and gait velocity with rhythmic feedback generated in real-time
Willems, et al. [173]	10 (67.2±9.1)	4	Step frequency, gait speed, stride length & double support (%) phase	Pre-test, gait performance at 0%, -20%, -10%, +10%, +20% of RAC (randomized), post-test	RAC at 0%, -20%, -10%, +10%, +20% preferred cadence	Significant effect of RAC on cadence, gait speed, with 0%, -10%, +10%, +20% pacing of RAC No significant effects on double limb support, stride length
Baker, et al. [101]	7F, 4M (71.5±2.5)	6	Gait speed, step amplitude & step frequency	Pre-test, functional gait performance with/without RAC -10% of preferred cadence, attentional cue instructions "try to take big steps", together "take a big step with the beat", & with/without a dual-task (a tray with 2 cups of water on top), post-test	RAC at -10% of preferred cadence	Significant effect of RAC & attentional cue "big steps with beat" on step frequency in gait speed (single-task only), step amplitude, step frequency in Ct in both single & dual-task conditions Non-significant effects on gait speed, step amplitude & step frequency with RAC only. Effects not evitable once the RAC was removed, in post-test
Rochester, et al. [94]	4F, 6M (63.5±7)	6	Step length, step frequency, walking speed, time duration & cadence	Complex functional walking & sitting task under single & dual-motor task (carrying a tray) condition with/without RAC	RAC generated per preferred speed of patients.	No effects of RAC on gait speed, step length & cadence under single/dual-task conditions. However, reduction in cadence under dual-task conditions with RAC
Thaut, et al. [174]	10F, 6M (25-40)	4	Stride symmetry, stride duration & EMG amplitude variability (Gastrocnemius)	Gait performance tested with/without RAC 3 times for 5 weeks	RAC at 4/4-time signature (1^st^ & 3^rd^ beat accentuated by tambourine beat, 70dB) at preferred cadence, at slower, faster than preferred cadence	Significant enhancement in stride rhythmicity between right & left limb with RAC Significantly delayed & shortened onset of gastrocnemius EMG activity with RAC Significant reduction in EMG variability of gastrocnemius muscle with RAC Significantly enhanced integrated amplitude ratios for gastrocnemius EMG activity
McIntosh, et al. [175]	6F, 4M (72±5)	4	Gait velocity, stride length, cadence & cadence-auditory stimulus synchronization	Gait performance by participants with pre-test, with & without RAC at +10% of preferred cadence, post-test	RAC at 0%, +10% of preferred cadence	Significant enhancement in gait velocity and cadence with RAC Enhancement in stride length. No effect on gait symmetry

F: Female, M: Male, Exp: Experimental group, Ct: Control group, RAC: Rhythmic auditory cueing, DFA: Detrended Fluctual Analysis, PD: Parkinson’s disease, EMG: Electromyography, IC: Isosynchronous cueing, bpm: beats per minute.

### Participants

A total of 854 participants were analyzed in the incorporated studies. Studies were then categorized into sub-groups for evaluating young and elderly participants. Three studies compared the effects of rhythmic auditory cueing amongst young and elderly participants. Eighteen studies evaluated elderly participants (68±5.6 years). A total of 355 participants were evaluated (235 females/100 males). Two studies did not specify the gender of the participants. All the studies evaluated a mixed gender sample size. Nineteen studies evaluated young participants (26.8±6 years). A total of 499 participants were evaluated (215 females/248 males). Two studies did not specify the gender of the included participants [91, 92]. Only one study evaluated a non-mixed gender sample i.e. only females [93]. Descriptive statistics relating to the age (mean ± standard deviation) of the participants were tabulated across the studies (Table 1).

### Risk of bias

**Figure 2.**
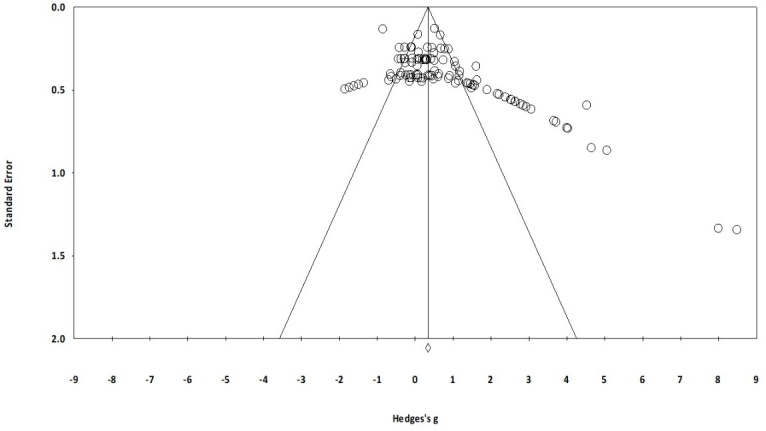
Funnel plot for Hedge’s g & standardized effect for each effect in the meta-analysis Each of the effect is represented in the plot as a circle. Funnel boundaries represent area where 95% of the effects are expected to abstain if there were no publication bias. The vertical line represents mean standardized effect of zero. Absence of publication bias is represented when the effects should be equally dispersed on either side of the line.

**Figure 3.**
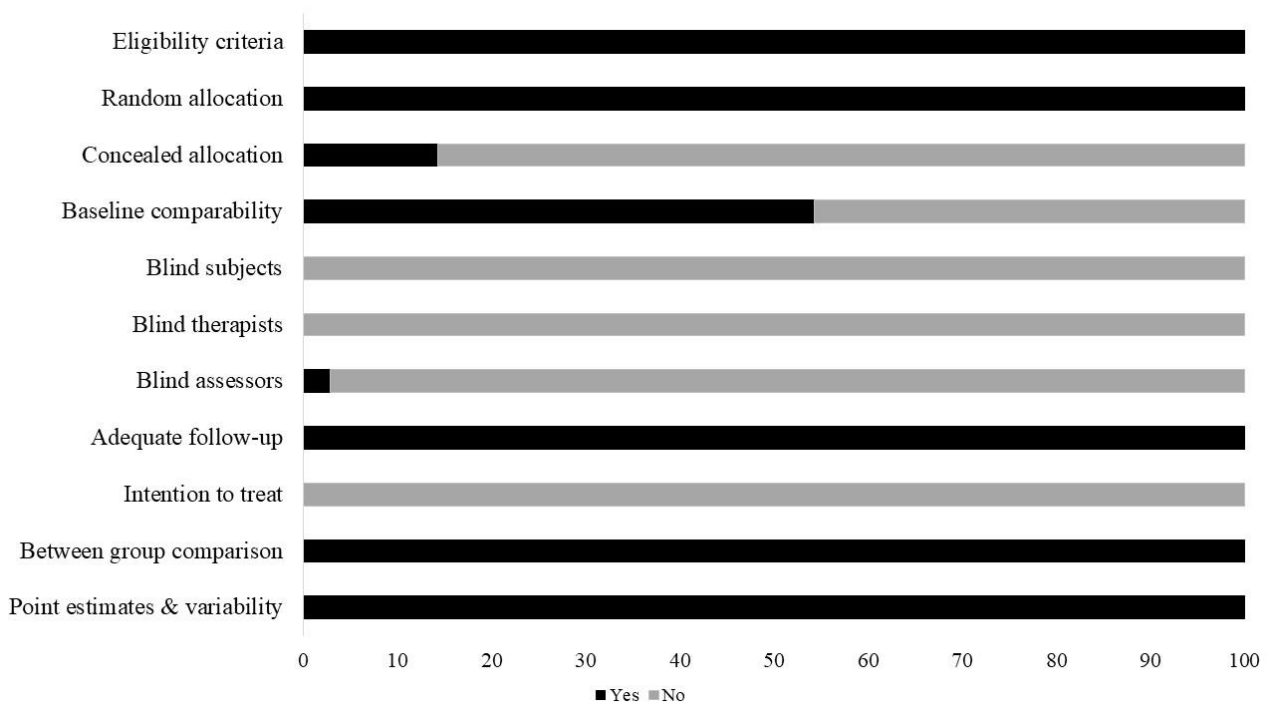
Risk of bias across studies.

The review included studies scoring ≥4 on PEDro to reduce the incidence of biasing. Moreover, the limitation of research protocols to be included in the review was limited to gold standard randomized controlled trials, cluster randomized controlled trials and controlled clinical trials. The individual scores attained by the studies using the PEDro scale have been reported (Table 1, Supplementary table 2). The average PEDro score for the fifty included studies was computed to be 4.7 out of 10, indicating fair-quality of the overall studies. One study scored 8, four scored 6, fourteen studies scored 5, and sixteen studies scored 4. Publication bias was analyzed by plotting a Hedge’s g against standard error (Fig. 2). Asymmetries concerning mean in the funnel plot might suggest bias (either positive or negative), in which case results are published. Risk of bias across the studies has been demonstrated in Fig. 3.

### Meta-Analysis

#### Outcomes

The results suggest evidence for a positive impact of rhythmic auditory cueing on spatiotemporal gait parameters amongst both young and elderly participants. In the included thirty-four studies, thirty studies reported significant enhancements, two studies reported enhancements (p>0.05) [94, 95], and two studies reported significant reduction in gait parameters with rhythmic auditory cueing [96, 97].

#### Meta-analysis report

The evaluation of research studies via meta-analysis requires strict inclusion criteria to efficiently limit the heterogeneity [98]. However, among the pooled group of studies post strict inclusion criteria, some amount of unexplained heterogeneity was still observed. Sub-group analysis was then performed for identical studies to evaluate the cause of heterogeneity. The evaluated parameters were the spatio-temporal gait parameters such as, cadence, stride length, gait velocity, coefficient of variability for stride time and stride length. The effects of fast/slow tempo on gait parameters in the included studies was determined by keeping the patient’s preferred cadence as reference. Analyses were also conducted to evaluate the effects of dual-task conditions, presence of instructions, and different tempo at which rhythmic auditory cueing was provided on gait parameters. We included a generalized group analysis first combined for all the pooled studies. A separate analysis in addition to clinical controlled trials was performed for high quality randomized controlled trails, for allowing a better interpretation of the direction and magnitude of effects. The main reason for not including the statistical approach within the studies was due to major differences in between assessment methods and lack of descriptive statistics within the manuscript. However, data was not received even after contacting the respective corresponding authors.

### Gait velocity

**Figure 4.**
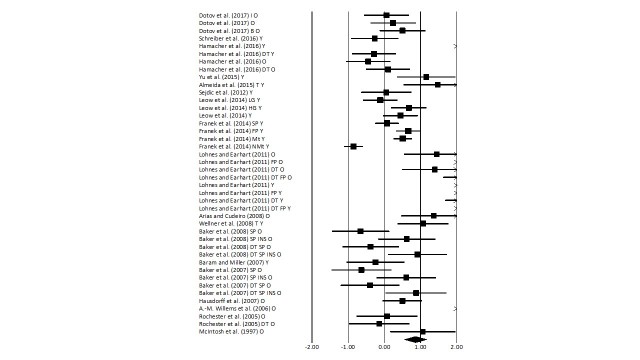
Forest plot illustrating individual studies evaluating the effects of rhythmic auditory cueing on gait velocity among healthy young and elderly participants A negative effect size indicated reduction in gait velocity; a positive effect size indicated enhancement in gait velocity. Weighted effect sizes; Hedge’s g (boxes) and 95% C.I (whiskers) are presented, demonstrating repositioning errors for individual studies. The (Diamond) represents pooled effect sizes and 95% CI. A negative mean difference indicates a favorable outcome for control groups; a positive mean difference indicates a favorable outcome for experimental groups. (O: Old, Y: Young, FP: Fast paced, SP: Slow paced, DT: Dual-task, I: Isosynchronous, B: Biological variability, LG: Low groove, HG: High groove, INS: Instructions, Mt: Motivating feedback, NMt: Non-motivating feedback).

The meta-analysis on healthy patients revealed (Fig. 4) a *large* effect size in positive domain with moderate heterogeneity (Hedge’s g: 0.85, 95% CI: 0.55 to 1.16, I^2^: 57.9%, p<0.01). Further, sub-group analysis was performed by dividing the groups in only young/elderly participants.

Young: The analysis for young participants performing gait with rhythmic auditory cueing revealed (Supplementary Fig. 1) beneficial effects with *large* effect and substantial heterogeneity (g: 0.92, 95% C.I: 0.42 to 1.41, I^2^: 93.2%, p<0.01). Further, sub-group analysis with non-modulated rhythmic auditory cueing (Supplementary Fig. 2), under a single task condition, revealed a *large* effect size with substantial heterogeneity (Hedge’s g: 1.24, 95% CI: 0.4 to 2, I^2^: 90.5%, p<0.01). The heterogeneity here could be attributed to different interventions utilized by studies. Wellner, et al. [91] for instance, utilized robot assisted gait, and Almeida, et al. [92] analyzed treadmill gait. Moreover, different measures of rhythmic auditory cueing were utilized by [99], as the study reported generation of rhythmic patterns by converting the foot strike patterns to rhythmic pattern in real-time.

Further, analysis with fast paced stimuli revealed (Supplementary Fig. 3) *large* effect size with substantial heterogeneity (g: 1.17, 95% C.I: 0.38 to 1.96, I^2^: 91.4%, p<0.01). Likewise, slow paced stimuli revealed (Supplementary Fig. 4) reduction in gait velocity parameters with *medium* effect size and substantial heterogeneity (g: -0.3, 95% C.I: 90.4%, I^2^: 90.4%, p<0.01). Here as well, the heterogeneity could be attributed to the type of entrainment used, for instance, low groove, non-motivating cueing and slow cueing were paired together and vice versa for the fast-paced stimuli. These stimuli differ in terms of emotional and expressiveness components, which might be considerably different from each other [68].

Dual task performance with auditory cueing in young participants with/without instructions to walk fast revealed (Supplementary Fig. 5) *large* effect size with substantial heterogeneity (g: 0.81, 95% C.I: 0.3-1.3, I^2^: 95.8%, p<0.01). Further, performance under pure dual-task conditions without any instructions revealed a *medium* positive effect size with substantial heterogeneity (g: 0.38, 95% C.I: -0.16 to 0.94, I^2^: 95%, p<0.01). Here, heterogeneity could be attributed to differential complexities of dual tasks incorporated within the studies, which in published literature have shown to portray different effects on motor performance [8].

**Figure 5.**
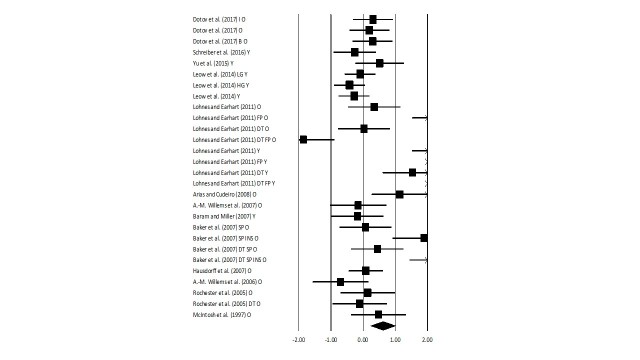
Forest plot illustrating individual studies evaluating the effects of rhythmic auditory cueing on stride length among healthy young and elderly participants A negative effect size indicated reduction in stride length; a positive effect size indicated enhancement in stride length. Weighted effect sizes; Hedge’s g (boxes) and 95% C.I (whiskers) are presented, demonstrating repositioning errors for individual studies. The (Diamond) represents pooled effect sizes and 95% CI. A negative mean difference indicates a favorable outcome for control groups; a positive mean difference indicates a favorable outcome for experimental groups. (O: Old, Y: Young, FP: Fast paced, SP: Slow paced, DT: Dual-task, I: Isosynchronous, B: Biological variability, LG: Low groove, HG: High groove, INS: Instructions, Mt: Motivating feedback, NMt: Non-motivating feedback).

Old: The analysis for old participants performing gait with rhythmic auditory cueing revealed (Supplementary Fig. 6) beneficial effects with *medium* effect and substantial heterogeneity (g: 0.68, 95% C.I: 0.28 to 1, I^2^: 81%, p<0.01). Further, sub-group analysis with non-modulated rhythmic auditory cueing revealed (Supplementary Fig. 7), under a single task condition, revealed a *medium* effect size with substantial heterogeneity (Hedge’s g: 0.73, 95% CI: 0.2 to 1.2, I^2^: 80.2%, p<0.01). Here, Dotov, Bayard, de Cock, Geny, Driss, Garrigue, Bardy and Dalla Bella [100] evaluated the effectiveness of feedbacks which were isosynchronous, and with/without biological variability. Possibly, the heterogeneity in the sub-group analysis could be attributed to the differential cueing utilized. Further, only one study analyzed the effects of fast paced stimuli amongst elderly and further couldn’t be included in sub-group analysis [67]. Slow paced stimuli, with/without verbal instructions revealed (Supplementary Fig. 8) enhancements in gait velocity parameters with *small* effect size and negligible heterogeneity (g: 0.25, 95% C.I: -0.49 to 1, I^2^: 0%, p>0.05). Additional, sub-group analysis revealed a considerable effect of verbal instructions over gait velocity i.e. analysis for performance without verbal instructions revealed a negative *medium* effect size with negligible heterogeneity (g: -0.4, 95% C.I: -0.98 to 0.18, I^2^: 0%, p>0.05), and including verbal instructions revealed a positive *large* effect size with negligible heterogeneity (g: 0.92, 95% C.I: 0.32 to 1.5, I^2^: 0%, p>0.05). Dual task performance with auditory cueing in elderly participants with/without instructions to walk fast revealed (Supplementary Fig. 9) a *medium* positive effect size with substantial heterogeneity (g: 0.58, 95% C.I: -0.05 to 1.2, I^2^: 79.2%, p>0.05). Performing under non-modulated rhythmic auditory cueing without any instructions with dual task revealed (Supplementary Fig. 10) a *medium* positive effect size (g: 0.43, 95% C.I: -0.44 to 1.3, I^2^: 13.4%, p>0.05) with negligible heterogeneity.

### Stride length

The meta-analysis on healthy patients revealed (Fig. 5) a *medium* effect size in positive domain with substantial heterogeneity (Hedge’s g: 0.61, 95% CI: 0.23 to 1, I^2^: 58.8%, p<0.05). Further, sub-group analysis was performed by dividing the groups in only young/elderly participants.

Young: The analysis for young participants performing gait with rhythmic auditory cueing revealed (Supplementary Fig. 11) beneficial effects with *large* effect and substantial heterogeneity (g: 1.2, 95% C.I: 0.38 to 2.85, I^2^: 92%, p<0.01). Further, sub-group analysis with non-modulated rhythmic auditory cueing revealed (Supplementary Fig. 12), under a single task condition, revealed a *large* effect size with substantial heterogeneity (Hedge’s g: 0.81, 95% CI: -0.5 to 1.7, I^2^: 88%, p<0.01). Further, analysis with fast paced stimuli revealed *small* effect size with substantial heterogeneity (g: -0.01, 95% C.I: -0.4 to 0.4, I^2^: 92.5%, p<0.01). The heterogeneity as stated before could be attributed to differential rhythmic stimuli utilized by studies. Moreover, none of the studies analyzing a slow-paced stimulus evaluated stride length. Dual task performance was analyzed in only one included study. Therefore, no further analysis could be carried out to evaluate the effects of higher information processing constraints on stride length.

**Figure 6.**
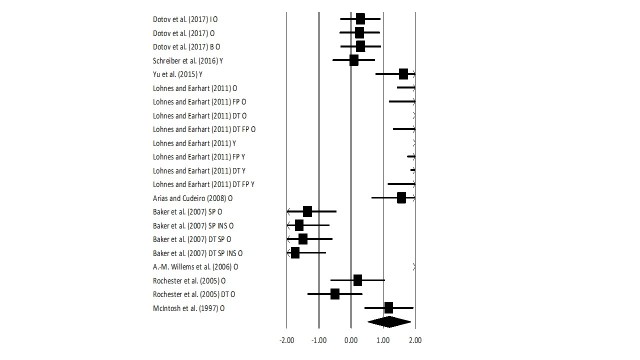
Forest plot illustrating individual studies evaluating the effects of rhythmic auditory cueing on cadence among healthy young and elderly participants A negative effect size indicated reduction in step frequency; a positive effect size indicated enhancement in step frequency. Weighted effect sizes; Hedge’s g (boxes) and 95% C.I (whiskers) are presented, demonstrating repositioning errors for individual studies. The (Diamond) represents pooled effect sizes and 95% CI. A negative mean difference indicates a favorable outcome for control groups; a positive mean difference indicates a favorable outcome for experimental groups. (O: Old, Y: Young, FP: Fast paced, SP: Slow paced, DT: Dual-task, I: Isosynchronous, B: Biological variability, LG: Low groove, HG: High groove, INS: Instructions, Mt: Motivating feedback, NMt: Non-motivating feedback)

Old: The analysis for old participants performing gait with rhythmic auditory cueing revealed (Supplementary Fig. 13) beneficial effects with *medium* effect and substantial heterogeneity (g: 0.39, 95% C.I: -0.01 to 0.78, I^2^: 77%, p<0.01). Further, sub-group analysis with non-modulated rhythmic auditory cueing revealed (Supplementary Fig. 14), under a single task condition, revealed a *small* effect size with negligible heterogeneity (Hedge’s g: 0.22, 95% CI: -0.03 to 0.46, I^2^: 10.5%, p>0.05). Further, one study each analyzed the effects of fast, slow paced stimuli amongst elderly and further couldn’t be included in sub-group analysis [67, 101]. Dual task performance with auditory cueing in elderly participants was analyzed amongst two studies [67, 94], a *small* effect size with negligible heterogeneity (g: -0.03, 95% C.I: -0.64 to 0.56, I^2^: 0%, p>0.05).

### Cadence

The meta-analysis on healthy patients revealed (Fig. 6) a *large* effect size in positive domain with moderate heterogeneity (Hedge’s g: 1.2, 95% CI: 0.51 to 1.8, I^2^: 41.9%, p<0.01). Further, sub-group analysis was performed by dividing the groups in only young/elderly participants.

Young: Further, sub-group analysis with non-modulated rhythmic auditory cueing revealed (Supplementary Fig. 15), under a single task condition, revealed a *large* effect size with substantial heterogeneity (Hedge’s g: 1.76, 95% CI: -0.29 to 3.8, I^2^: 93.2%, p<0.01). Only one study performed [67], rhythmic auditory cueing with fast pace and no study analyzed the effects with slow paced stimulus. Therefore, no additional analysis was carried out. Dual task performance was analyzed in only one included study. Therefore, no further analysis could be carried out to evaluate the effects of higher information processing constraints on cadence.

Old: The analysis for old participants performing gait with rhythmic auditory cueing revealed (Supplementary Fig. 16) beneficial effects with *medium* effect and substantial heterogeneity (g: 0.78, 95% C.I: 0.01 to 1.54, I^2^: 91.5%, p<0.01). Sub-group analysis with non-modulated rhythmic auditory (Supplementary Fig. 17), under a single task condition, revealed a *large* effect size with substantial heterogeneity (Hedge’s g: 1.02, 95% CI: 0.19 to 1.84, I^2^: 88.6%, p<0.01). Further, one study each analyzed the effects of fast, slow paced stimuli amongst elderly and further couldn’t be included in sub-group analysis [67, 101]. Dual task performance with auditory cueing in elderly participants was analyzed amongst two studies [67, 94], a *medium* effect size with substantial heterogeneity (g: 0.68, 95% C.I: -0.03 to -1.41, I^2^: 96%, p<0.01).

### Coefficient of variability stride time

Analysis of coefficient of variability for stride time revealed (Supplementary Fig. 18) a *small* effect in positive domain with substantial heterogeneity (g: 0.21, 95% C.I: -0.42 to 0.85, I^2^: 67.7%, p<0.05). Further, in a sub-group analysis for only old participants revealed a *medium* effect size in positive domain with substantial heterogeneity (g: 0.4, 95% C.I: -0.33 to 1.13, I^2^: 63%, p<0.05) [102-104].

### Coefficient of variability stride length

Analysis of coefficient of variability for stride length revealed (Supplementary Fig. 19) a *medium* effect in positive domain with moderate heterogeneity (g: 0.76, 95% C.I: 0.43 to 1.1, I^2^: 48.7%, p>0.05) [102, 104, 105]. Further, in a sub-group analysis for only young participants with non-modulated rhythmic auditory cueing revealed a *medium* effect size in positive domain with negligible heterogeneity (g: 0.47, 95% C.I: -0.09 to 0.85, I^2^: 4.7%, p>0.05) [104, 105]. Likewise, for only old participants a *large* effect size in positive domain with negligible heterogeneity (g: 1.01, 95% C.I: -0.17 to 2.2, I^2^: 0%, p>0.05) was observed [102, 104].

## DISCUSSION

The primary objective of this present systematic review and meta-analysis was to synthesize the current state of knowledge for effects that rhythmic auditory cueing might lay over aging gait. Out of thirty-four included studies, 88% studies reported beneficial effects of rhythmic auditory cueing on primary spatiotemporal gait parameters.

Typically, spatiotemporal parameters of gait worsen with age [19, 106]. Callisaya, Beare, Phan, Blizzard, Thrift, Chen and Srikanth [107], studied age associated decline in brain structure with gait performance, and linked a reduction in gait velocity, stride length, cadence with white matter atrophy, lesions, hippocampal atrophy, and gray matter atrophy with cerebral infarcts, respectively [107, 108]. Moreover, research suggests that degenerative changes in the fronto-striatal circuits might add increasing bi-directional stress on automated control for posture, gait and cognitive processing [109-111]. Possibly, explaining the loss of gait rhythmicity in elderly (see also, Nombela, et al. [56]). Likewise, increased energy expenditure [108], weak musculoskeletal structure associated variability in muscle contraction, and force production add towards the woes [112]. The current meta-analysis reported enhancements in gait velocity (g: 0.68), stride length (0.39) and cadence (0.78), post application of rhythmic auditory cueing in elderly population groups. Likewise, beneficial effects of rhythmic auditory cueing were also observed in gait amongst younger population groups.

Several mechanisms have been suggested to ascertain the beneficial effects of rhythmic auditory cueing. Rizzo, Raghavan, McCrery, Oh-Park and Verghese [113] for instance, speculated that auditory entrainment while performing gait might act as an efficient distractor. In addition, the auditory entrainment might also have aided in reducing the errors while executing the gait [114, 115]. Possibly, by acting as an external guidance for “heel-contact” and “push-off” timings. Moreover, application of auditory entrainment is believed to allow enhancement in gait performance by bypassing or facilitating the degenerated basal ganglia-motor loop via alternative pathways [116-118]. Cunnington, Iansek, Bradshaw and Phillips [119] suggested that the external stimulation by entrainment might surpass deficient pallidal-cortical projections, and can directly serve an input supplementary motor area, thereby reducing the onset of motor deficit and aiding in performance. Moreover, the external cueing has shown to allow modulation of neuromagnetic β oscillations in auditory cortex, cerebellum, inferior frontal gyrus, somatosensory area and sensorimotor cortex [120], and reduce hemispheric asymmetry [121]. Neuroimaging studies reveal enhance activation in inferior colliculi [122], cerebellum, brainstem [117, 123], sensorimotor cortex [124, 125], further instigating cortico-cerebellar network re-organization [126]. Another crucial factor that considerably influences the aging gait is “change in tempo”. Neurophysiological analysis suggests, increased neuronal activation in fronto-occipital networks [127], and excitability of the spinal motor neurons by reticulospinal pathways, with fast-paced entrainment. A paced-stimuli is thought to reduce the response time, limit the stagnating effects of constant entrainment over fractal scaling of stride times from healthy 1/f structure [128-130], and optimizing the velocity and acceleration profiles of joint motions by scaling movement time [59].

The present-meta-analysis also observed enhancements in the spatiotemporal parameters while performing dual-tasks, for both age groups. According to literature, dual-task performance predisposes to gait instability and falls by increasing cognitive motor interferences, across age groups [8, 131-133]. Interpretations from our results suggest that rhythmic auditory cueing counteracts cognitive constraints imposed by cognitively demanding dual-tasks such as carrying a tray and that this cueing might be useful in counteracting fall while carrying out activities of daily living [8]. Lohnes and Earhart [67], suggested that co-performance of dual-tasks with rhythmic auditory cueing might allow enhancements (or even stability) in performance, by possibly freeing up cognitive resources for dual-task performance. The authors also mentioned the influence of task complexity across age groups. Possibly, the freed up cognitive resources might not be sufficient especially in elderly to perform complex dual-tasks, such as coin transfer [134], and sentence reciting tasks [135]. This might possibly explain the reduced dual tasks costs on gait performance in young participants. In addition, the enhanced performance could also be attributed as to how the participants might perceive the auditory entrainment based on their cognitive capabilities. Wittwer, Webster and Hill [136], and Thaut, Miltner, Lange, Hurt and Hoemberg [137], suggested a strong relationship in between the cognitive capabilities and the ability to interpret and discern the structure of a beat. Thereby, suggesting a better rhythmic perception and interpretation by younger population groups as compared to their older counterparts.

Moreover, the progressive degradation of neuromuscular structures with aging has further been suggested to alleviate the threshold for action relevant acoustic input [138]. To counteract this deficiency use of ecologically valid acoustic feedback has been suggested [138]. The ecologically valid action related sounds might enhance saliency of sensory information concerning spatiotemporal information, thereby aiding in movement execution [100, 138-141]. This was also demonstrated by Dotov, et al. [100], here the authors demonstrated beneficial effects in parkinsonian and healthy gait parameters with biologically variable rhythmic auditory cueing as compared to isosynchronous cueing. Moreover, recent research has also revealed the possibilities of including emotional [113], motivational [68], and expressiveness [142], component in auditory entrainment to portray differential effects on gait parameters. Unfortunately, lack of pertinent, repeatable literature concerning the specific type of modified auditory feedback makes it difficult to interpret, as to which type of feedback might be most optimal, and for which age groups. We suggest future studies to replicate data concerning the use of ecological auditory entrainment across different age groups, to allow a reliable interpretation, which could then be included in gait rehabilitation protocols. Moreover, we also suggest future researchers to analyze the “entrainment effects” while multitasking in high-stress situations pertinent to modern day scenarios (for example, walking and texting, listening to music while crossing a traffic light).

This current meta-analysis also reported an increase in coefficient of stride-time and length variability in elderly participants with rhythmic auditory cueing. Based, on the published literature initial increase in variability during learning paradigm is efficient for improving gait performance [143]. Here, interpretations could possibly be drawn from “dynamic system theory” [144]. The theory suggests that a biological system might allow variability to identify and self-organize the most stable and viable outcome [144, 145]. Thereby, interpretations could be made for regulating gait amongst young and elderly population groups to regulate gait when passing through fall-prone environments [41]. The present meta-analysis did not evaluate the the influence of gait training with rhythmic auditory cueing on ageing gait. Whereas, training regimes with auditory entrainment have demonstrated reduced variability in parkinsonism [101, 146], and stroke [126]. We suggest future research to address this gap in the literature and evaluate the effects of long term training with rhythmic auditory cueing on aging gait.

Finally, we believe that the benefits of auditory entrainment might surpass that of co-treatment techniques (for instance, biofeedback, virtual reality, physiotherapy etc.) because of its economical nature, and high viability [77, 78]. The rhythmic entrainment factor could be utilized with music in rehabilitation, day to day lives. This could allow benefits in both psycho-physiological domains [147-151]. For instance, improving stress, mediating arousal, emotions, internal motivation, memory, attention, executive functions [152], power [153], and endurance [154]. Moreover, it is important to consider that the retention of enhancements in gait parameters relies not only on the training received in the clinic but also depends largely on how much the patient follows the treatment protocol at home. Lim, et al. [13] for instance, reported enhancement in parkinsonian gait activity to 35 minutes per day (qualifying the 30 minutes criteria by WHO [155]). We believe that delivering this type of home-based intervention could possibly be beneficial for people lacking proper exposure to medical interventions in developing countries [156]. For instance, a booming number of smartphone devices in developing countries [157], can be used as a delivery tool while using a simple metronome app such as, Walkmate [129], or Listenmee [158], which with proper medical guidance might allow curbing the motor deficits associated with aging [159]. We also suggest the use of rhythmic auditory cueing as an adjunct to other rehabilitation strategies, for instance, dance, tai-chi, aerobics, as it might enhance the rehabilitation progress by focusing on both psycho-physiological components.

To the best of our knowledge, this present review for the first time analyzed the effects of auditory entrainment on aging gait. The present findings are in agreement with systematic reviews and meta-analysis carried out to analyze auditory entrainment effect on stroke [66], cerebral palsy [160], and parkinsonism [57, 161]. In conclusion, this review strongly suggests the incorporation of rhythmic auditory cueing for enhancing gait performance with aging gait. The results from the meta-analysis also direct towards the possible use of auditory entrainment to reduce the incidence of falls in high-stress situations.

## Supplemental data

Supplemental data are available online at www.aginganddisease.org/EN/10.14336/AD.2017.1031.
