# Supplementary material for: Effect of Rhythmic Auditory Cueing on Aging Gait: A Systematic Review and Meta-Analysis
Source: Aging Dis. 2018 Oct 1;9(5):901–23. doi: 10.14336/AD.2017.1031 (PMC6147584; doi:10.14336/AD.2017.1031)
Supplement: Supplementary file 1 — Supplemental data are available online at www.aginganddisease.org/EN/10.14336/AD.2017.1031. [file AD-9-5-901-s.pdf]

SUPPLEMENTARY DATA

Effect of Rhythmic Auditory Cueing on Aging Gait: A Systematic Review and Meta-Analysis

Shashank Ghai[1, *], Ishan Ghai[2], Alfred O. Effenberg[1]

[1]Institute for Sports Science, Leibniz University Hannover, Germany
[2]School of Life Sciences, Jacobs University Bremen, Germany

© 2017. Ghai S et al. Published online at http://www.aginganddisease.org/EN/10.14336/AD.2017.1031

SUPPLEMENTARY DATA

Supplemental Table 1. Sample search strategy EMBASE.

**DATABSE**		**EMBASE**
**DATE**		**10/07/2017**
**STRATEGY**		**#1 AND #2 AND #3 AND #4 AND #5**
	**#1**	(‘rhythmic auditory feedback’ OR ‘rhythmic auditory cueing’ OR ‘rhythmic acoustic feedback’ OR ‘rhythmic auditory entrainment’ OR ‘metronome feedback’ OR ‘metronome’ OR ‘rhythmic metronome feedback’ OR ‘acoustic stimulus’ OR ‘acoustic feedback’ OR ‘acoustic cueing’ OR ‘external stimuli’ OR ‘external feedback’ OR ‘external cueing’ OR ‘music therapy’ OR ‘Neurological music therapy’ OR ‘tempo’ OR ‘beat’ OR ‘rhythm’ OR ‘RAC’ OR ‘NMT’)/de OR (rhythmic auditory feedback OR rhythmic auditory cueing OR rhythmic acoustic feedback OR rhythmic auditory entrainment OR metronome feedback OR metronome OR rhythmic metronome feedback OR acoustic stimulus OR acoustic feedback OR acoustic cueing OR external stimuli OR external feedback OR external cueing OR music therapy OR Neurological music therapy OR tempo OR beat OR rhythm OR RAC OR NMT)ti,ab
	**#2**	(‘cognitive task’ OR ‘concurrent task’ OR ‘dual task’ OR ‘dual task’ OR ‘dual task paradigm’ OR ‘dual task paradigm’ OR ‘cognitive task training’ OR ‘dual task training’ OR ‘dual task training’)/de OR (cognitive task OR concurrent task OR dual task OR dual task OR dual task paradigm OR dual task paradigm OR cognitive task training OR dual task training OR dual task training’):ti,ab
	**#3**	(‘rehabilitation’ OR ‘treatment’ OR ‘rehab’ OR ‘management’ OR ‘therapy’ OR ‘physiotherapy’ OR ‘physical therapy’ OR ‘prevention’ OR ‘risk prevention’)/de OR (rehabilitation OR treatment OR rehab OR management OR therapy OR physiotherapy OR physical therapy OR prevention OR risk prevention);ti,ab
	**#4**	(‘age groups’ OR ‘adolescent’ OR ‘young’ OR ‘elderly’ OR ‘old’ AND (‘gender’ OR ‘male’ OR ‘female’)/de OR (age groups OR adolescent OR young OR elderly OR old AND (gender OR male OR female));ti;ab
	**#5**	clinical trial/exp OR (‘intervention study’ OR ‘cohort analysis’ OR ‘longitudinal study’ OR ‘cluster analysis’ OR ‘crossover trial’ OR ‘cluster analysis’ OR ‘randomized trial’ OR ‘major clinical study’)/de OR (longitudinal OR cohort OR crossover trial OR cluster analysis OR randomized trial OR clinical trial OR controlled trial);ti,ab

© 2017. Ghai S et al. Published online at http://www.aginganddisease.org/EN/10.14336/AD.2017.1031

SUPPLEMENTARY DATA

Supplemental Table 2. Individual Pedro scores.

Study	Total PEDRO	Point estimates & variability	Between group comparison	Intention to treat	Adequate follow-up	Blind assessors	Blind therapists	Blind subjects	Baseline comparability	Concealed allocation	Random allocation	Eligibility criteria
Dotov et al. (2017)	6	1	1	0	1	0	0	0	1	1	1	1
Maculewicz et al. (2016)	4	1	1	0	1	0	0	0	0	0	1	1
Terrier (2016)	4	1	1	0	1	0	0	0	0	0	1	1
Schreiber et al. (2016)	4	1	1	0	1	0	0	0	0	0	1	1
Hamacher et al. (2016)	5	1	1	0	1	0	0	0	1	0	1	1
Yu et al. (2015)	5	1	1	0	1	0	0	0	1	0	1	1
Almeida et al. (2015)	4	1	1	0	1	0	0	0	0	0	1	1
Kennel et al. (2015)	5	1	1	0	1	0	0	0	1	0	1	1
Roerdink et al. (2015)	5	1	1	0	1	0	0	0	1	0	1	1
Leow et al. (2014)	5	1	1	0	1	0	0	0	1	0	1	1
Franek et al. (2014)	4	1	1	0	1	0	0	0	0	0	1	1
Marmelat et al. (2014)	5	1	1	0	1	0	0	0	1	0	1	1
Hunt et al. (2014)	4	1	1	0	1	0	0	0	0	0	1	1
Wright et al. (2014)	5	1	1	0	1	0	0	0	1	0	1	1
Wittwer et al. (2013b)	4	1	1	0	1	0	0	0	0	0	1	1
Leman et al. (2013)	4	1	1	0	1	0	0	0	0	0	1	1
Bank et al. (2011)	5	1	1	0	1	0	0	0	1	0	1	1
Peper et al. (2012)	5	1	1	0	1	0	0	0	1	0	1	1
Sejdic et al. (2012)	5	1	1	0	1	0	0	0	1	0	1	1
Terrier and Dériaz (2012a)	4	1	1	0	1	0	0	0	0	0	1	1
Trombetti et al. (2011)	8	1	1	0	1	1	0	0	1	1	1	1
Roerdink et al. (2011)	5	1	1	0	1	0	0	0	1	0	1	1
Lohnes and	5	1	1	0	1	0	0	0	1	0	1	1

© 2017. Chen W et al. Published online at http://www.aginganddisease.org/EN/10.14336/AD.2018.0606

Earhart (2011)												
Baker et al. (2008)	6	1	1	0	1	0	0	0	1	1	1	1
Arias and Cudeiro (2008)	4	1	1	0	1	0	0	0	0	0	1	1
Wellner et al. (2008)	4	1	1	0	1	0	0	0	0	0	1	1
A.-M. Willems et al. (2007)	5	1	1	0	1	0	0	0	1	0	1	1
Baker et al. (2007)	6	1	1	0	1	0	0	0	1	1	1	1
Baram and Miller (2007)	4	1	1	0	1	0	0	0	0	0	1	1
Hausdorff et al. (2007)	5	1	1	0	1	0	0	0	1	0	1	1
A.-M. Willems et al. (2006)	4	1	1	0	1	0	0	0	0	0	1	1
Chen et al. (2006a)	4	1	1	0	1	0	0	0	0	0	1	1
Rochester et al. (2005)	6	1	1	0	1	0	0	0	1	1	1	1
McIntosh et al. (1997)	4	1	1	0	1	0	0	0	0	0	1	1
Thaut et al. (1992)	4	1	1	0	1	0	0	0	0	0	1	1

1: Point awarded, 0: Point not awarded

© 2017. Chen W et al. Published online at http://www.aginganddisease.org/EN/10.14336/AD.2018.0606

SUPPLEMENTARY DATA

Meta-analysis Supplemental Figures

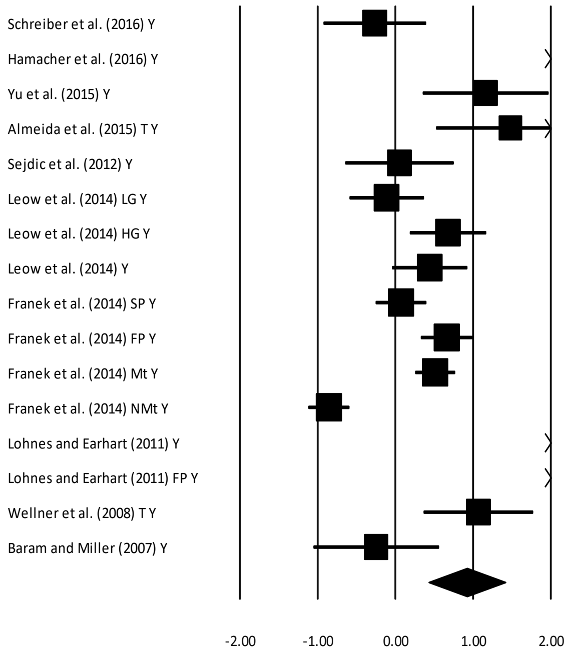

Supplemental Figure 1. Forest plot illustrating individual studies evaluating the effects of rhythmic auditory cueing on gait velocity among healthy young participants. A negative effect size indicated reduction in gait velocity; a positive effect size indicated enhancement in gait velocity. Weighted effect sizes; Hedge's g (boxes) and 95% C.I (whiskers) are presented, demonstrating repositioning errors for individual studies. The (Diamond) represents pooled effect sizes and 95% CI. A negative mean difference indicates a favorable outcome for control groups; a positive mean difference indicates a favorable outcome for experimental groups. (O: Old, Y: Young, FP: Fast paced, SP: Slow paced, DT: Dual-task, I: Isosynchronous, B: Biological variability, LG: Low groove, HG: High groove, INS: Instructions, Mt: Motivating feedback, NMt: Non-motivating feedback)

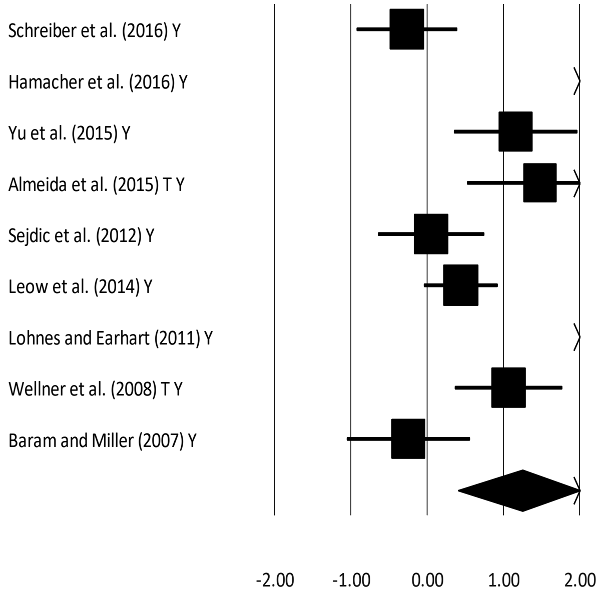

Supplemental Figure 2. Forest plot illustrating individual studies evaluating the effects of rhythmic auditory cueing on gait velocity among healthy young participants, un-modulated rhythmic auditory cueing. A negative effect size indicated reduction in gait velocity; a positive effect size indicated enhancement in gait velocity. Weighted effect sizes; Hedge's g (boxes) and 95% C.I (whiskers) are presented, demonstrating repositioning errors for individual studies. The (Diamond) represents pooled effect sizes and 95% CI. A negative mean difference indicates a favorable outcome for control groups; a positive mean difference indicates a favorable outcome for experimental groups. (O: Old, Y: Young, FP: Fast paced, SP: Slow paced, DT: Dual-task, I: Isosynchronous, B: Biological variability, LG: Low groove, HG: High groove, INS: Instructions, Mt: Motivating feedback, NMt: Non-motivating feedback)

© 2017. Chen W et al. Published online at http://www.aginganddisease.org/EN/10.14336/AD.2018.0606

SUPPLEMENTARY DATA

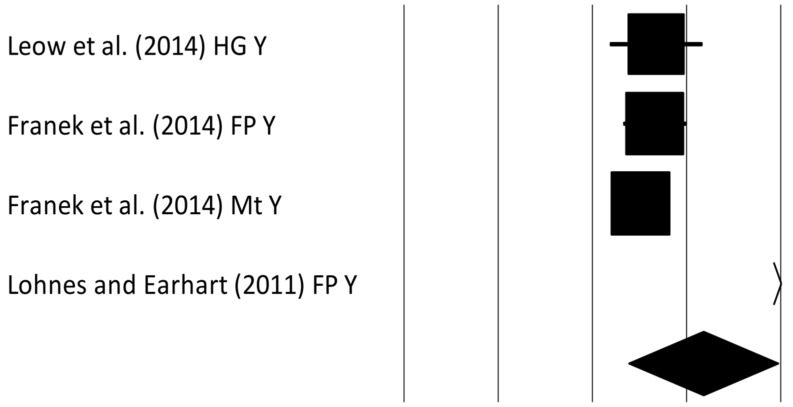

Supplemental Figure 4 Forest plot illustrating individual studies evaluating the effects of rhythmic auditory cueing on gait velocity among healthy young participants with fast paced modified stimuli (measured according to preferred cadence). A negative effect size indicated reduction in gait velocity; a positive effect size indicated enhancement in gait velocity. Weighted effect sizes; Hedge's g (boxes) and 95% C.I (whiskers) are presented, demonstrating repositioning errors for individual studies. The (Diamond) represents pooled effect sizes and 95% CI. A negative mean difference indicates a favorable outcome for control groups; a positive mean difference indicates a favorable outcome for experimental groups. (O: Old, Y: Young, FP: Fast paced, SP: Slow paced, DT: Dual-task, I: Isosynchronous, B: Biological variability, LG: Low groove, HG: High groove, INS: Instructions, Mt: Motivating feedback, NMt: Non-motivating feedback)

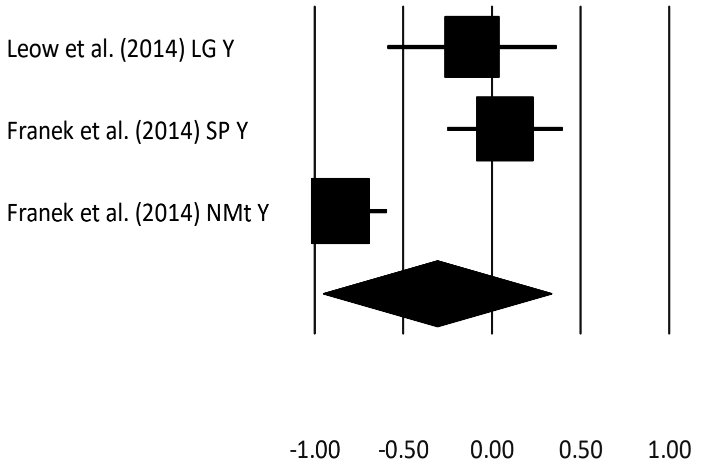

Supplemental Figure 3 Forest plot illustrating individual studies evaluating the effects of rhythmic auditory cueing on gait velocity among healthy young participants with slow paced modified stimuli (measured according to preferred cadence). A negative effect size indicated reduction in gait velocity; a positive effect size indicated enhancement in gait velocity. Weighted effect sizes; Hedge's g (boxes) and 95% C.I (whiskers) are presented, demonstrating repositioning errors for individual studies. The (Diamond) represents pooled effect sizes and 95% CI. A negative mean difference indicates a favorable outcome for control groups; a positive mean difference indicates a favorable outcome for experimental groups. (O: Old, Y: Young, FP: Fast paced, SP: Slow paced, DT: Dual-task, I: Isosynchronous, B: Biological variability, LG: Low groove, HG: High groove, INS: Instructions, Mt: Motivating feedback, NMt: Non-motivating feedback)

© 2017. Chen W et al. Published online at http://www.aginganddisease.org/EN/10.14336/AD.2018.0606

SUPPLEMENTARY DATA

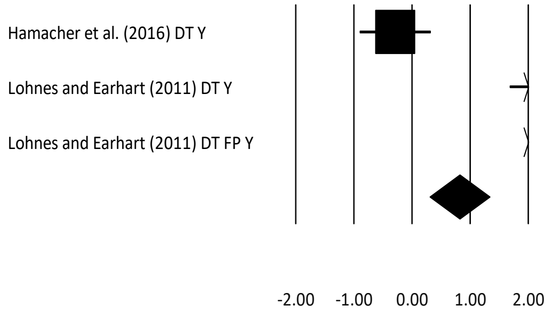

Supplemental Figure 5. Forest plot illustrating individual studies evaluating the effects of rhythmic auditory cueing on gait velocity among healthy young participants under dual-task conditions. A negative effect size indicated reduction in gait velocity; a positive effect size indicated enhancement in gait velocity. Weighted effect sizes; Hedge's g (boxes) and 95% C.I (whiskers) are presented, demonstrating repositioning errors for individual studies. The (Diamond) represents pooled effect sizes and 95% CI. A negative mean difference indicates a favorable outcome for control groups; a positive mean difference indicates a favorable outcome for experimental groups. (O: Old, Y: Young, FP: Fast paced, SP: Slow paced, DT: Dual-task, I: Isosynchronous, B: Biological variability, LG: Low groove, HG: High groove, INS: Instructions, Mt: Motivating feedback, NMt: Non-motivating feedback)

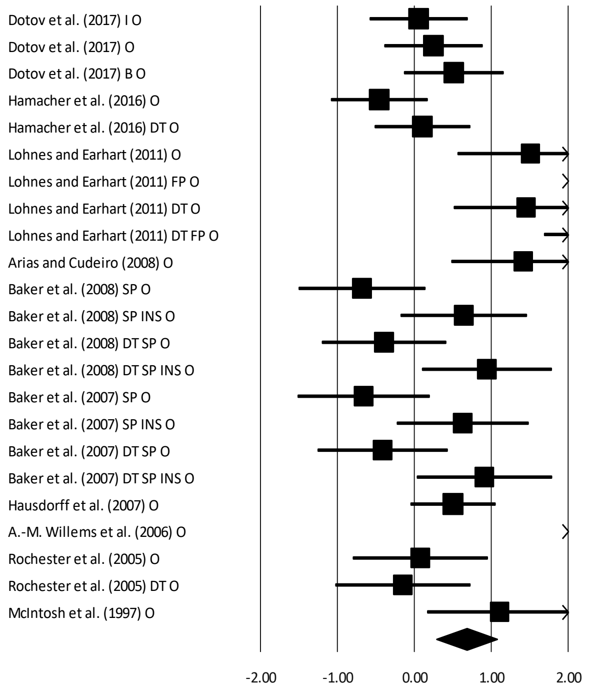

Supplemental Figure 6. Forest plot illustrating individual studies evaluating the effects of rhythmic auditory cueing on gait velocity among healthy old participants. A negative effect size indicated reduction in gait velocity; a positive effect size indicated enhancement in gait velocity. Weighted effect sizes; Hedge's g (boxes) and 95% C.I (whiskers) are presented, demonstrating repositioning errors for individual studies. The (Diamond) represents pooled effect sizes and 95% CI. A negative mean difference indicates a favorable outcome for control groups; a positive mean difference indicates a favorable outcome for experimental groups. (O: Old, Y: Young, FP: Fast paced, SP: Slow paced, DT: Dual-task, I: Isosynchronous, B: Biological variability, LG: Low groove, HG: High groove, INS: Instructions, Mt: Motivating feedback, NMt: Non-motivating feedback)

© 2017. Chen W et al. Published online at http://www.aginganddisease.org/EN/10.14336/AD.2018.0606

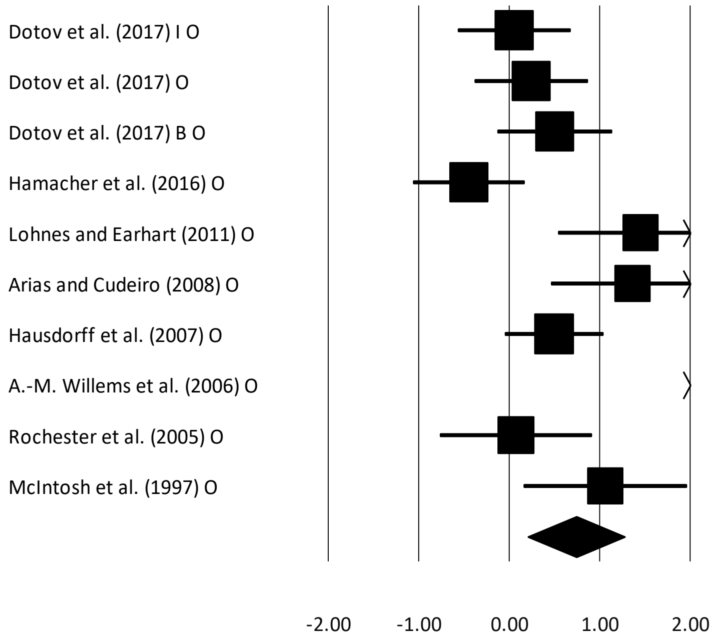

Supplemental Figure 7. Forest plot illustrating individual studies evaluating the effects of rhythmic auditory cueing on gait velocity among healthy old participants, un-modulated rhythmic auditory cueing. A negative effect size indicated reduction in gait velocity; a positive effect size indicated enhancement in gait velocity. Weighted effect sizes; Hedge's g (boxes) and 95% C.I (whiskers) are presented, demonstrating repositioning errors for individual studies. The (Diamond) represents pooled effect sizes and 95% CI. A negative mean difference indicates a favorable outcome for control groups; a positive mean difference indicates a favorable outcome for experimental groups. (O: Old, Y: Young, FP: Fast paced, SP: Slow paced, DT: Dual-task, I: Isosynchronous, B: Biological variability, LG: Low groove, HG: High groove, INS: Instructions, Mt: Motivating feedback, NMt: Non-motivating feedback)

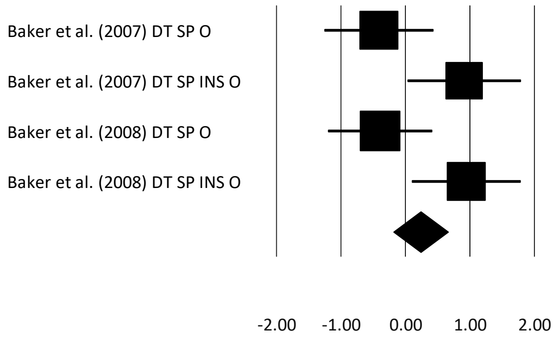

Supplemental Figure 8. Forest plot illustrating individual studies evaluating the effects of rhythmic auditory cueing on gait velocity among healthy old participants with slow paced modified stimuli (measured according to preferred cadence). A negative effect size indicated reduction in gait velocity; a positive effect size indicated enhancement in gait velocity. Weighted effect sizes; Hedge's g (boxes) and 95% C.I (whiskers) are presented, demonstrating repositioning errors for individual studies. The (Diamond) represents pooled effect sizes and 95% CI. A negative mean difference indicates a favorable outcome for control groups; a positive mean difference indicates a favorable outcome for experimental groups. (O: Old, Y: Young, FP: Fast paced, SP: Slow paced, DT: Dual-task, I: Isosynchronous, B: Biological variability, LG: Low groove, HG: High groove, INS: Instructions, Mt: Motivating feedback, NMt: Non-motivating feedback).

© 2017. Chen W et al. Published online at http://www.aginganddisease.org/EN/10.14336/AD.2018.0606

SUPPLEMENTARY DATA

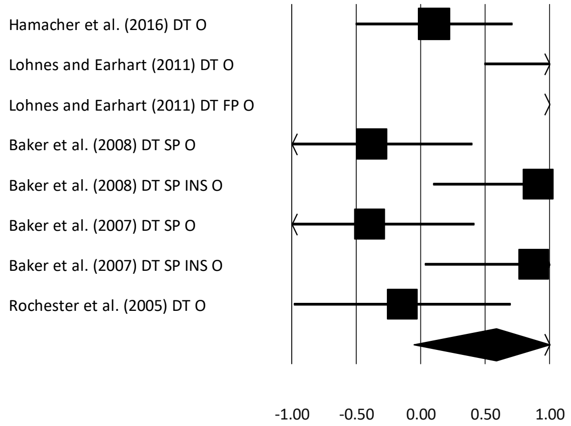

Supplemental Figure 9. Forest plot illustrating individual studies evaluating the effects of rhythmic auditory cueing on gait velocity among healthy old participants under dual-task conditions (fast & slow-paced cueing). A negative effect size indicated reduction in gait velocity; a positive effect size indicated enhancement in gait velocity. Weighted effect sizes; Hedge's g (boxes) and 95% C.I (whiskers) are presented, demonstrating repositioning errors for individual studies. The (Diamond) represents pooled effect sizes and 95% CI. A negative mean difference indicates a favorable outcome for control groups; a positive mean difference indicates a favorable outcome for experimental groups. (O: Old, Y: Young, FP: Fast paced, SP: Slow paced, DT: Dual-task, I: Isosynchronous, B: Biological variability, LG: Low groove, HG: High groove, INS: Instructions, Mt: Motivating feedback, NMt: Non-motivating feedback)

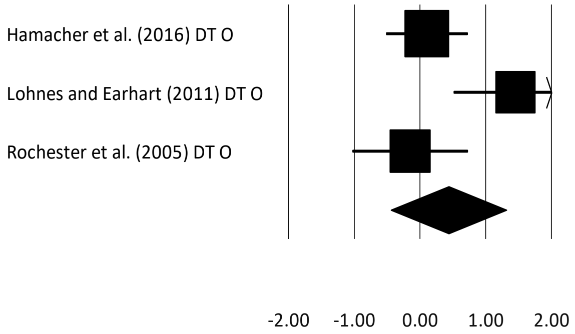

Supplemental Figure 10. Forest plot illustrating individual studies evaluating the effects of rhythmic auditory cueing on gait velocity among healthy old participants under dual-task conditions (un-modulated rhythmic auditory cueing). A negative effect size indicated reduction in gait velocity; a positive effect size indicated enhancement in gait velocity. Weighted effect sizes; Hedge's g (boxes) and 95% C.I (whiskers) are presented, demonstrating repositioning errors for individual studies. The (Diamond) represents pooled effect sizes and 95% CI. A negative mean difference indicates a favorable outcome for control groups; a positive mean difference indicates a favorable outcome for experimental groups. (O: Old, Y: Young, FP: Fast paced, SP: Slow paced, DT: Dual-task, I: Isosynchronous, B: Biological variability, LG: Low groove, HG: High groove, INS: Instructions, Mt: Motivating feedback, NMt: Non-motivating feedback)

© 2017. Chen W et al. Published online at http://www.aginganddisease.org/EN/10.14336/AD.2018.0606

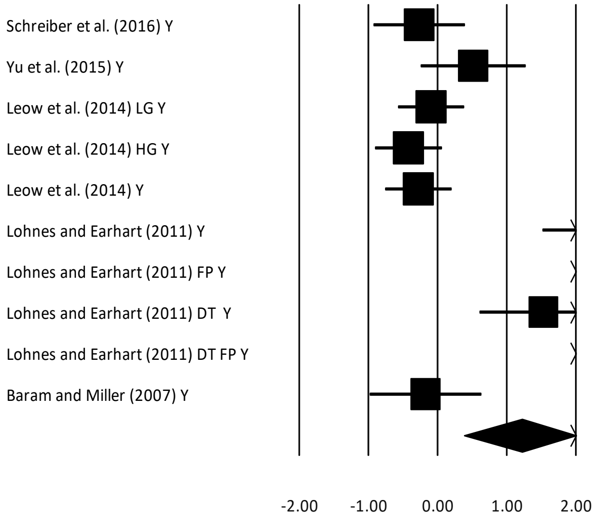

Supplemental Figure 11 Forest plot illustrating individual studies evaluating the effects of rhythmic auditory cueing on stride length among healthy young participants. A negative effect size indicated reduction in stride length; a positive effect size indicated enhancement in stride length. Weighted effect sizes; Hedge's g (boxes) and 95% C.I (whiskers) are presented, demonstrating repositioning errors for individual studies. The (Diamond) represents pooled effect sizes and 95% CI. A negative mean difference indicates a favorable outcome for control groups; a positive mean difference indicates a favorable outcome for experimental groups. (O: Old, Y: Young, FP: Fast paced, SP: Slow paced, DT: Dual-task, I: Isosynchronous, B: Biological variability, LG: Low groove, HG: High groove, INS: Instructions, Mt: Motivating feedback, NMt: Non-motivating feedback)

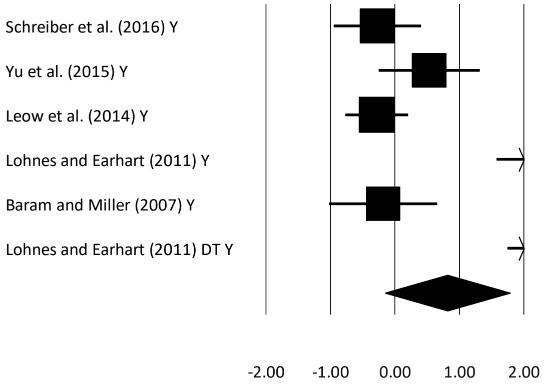

Supplemental Figure 12. Forest plot illustrating individual studies evaluating the effects of rhythmic auditory cueing on stride length among healthy young participants (non-modulated rhythmic auditory cueing). A negative effect size indicated reduction in stride length; a positive effect size indicated enhancement in stride length. Weighted effect sizes; Hedge's g (boxes) and 95% C.I (whiskers) are presented, demonstrating repositioning errors for individual studies. The (Diamond) represents pooled effect sizes and 95% CI. A negative mean difference indicates a favorable outcome for control groups; a positive mean difference indicates a favorable outcome for experimental groups. (O: Old, Y: Young, FP: Fast paced, SP: Slow paced, DT: Dual-task, I: Isosynchronous, B: Biological variability, LG: Low groove, HG: High groove, INS: Instructions, Mt: Motivating feedback, NMt: Non-motivating feedback)

© 2017. Chen W et al. Published online at http://www.aginganddisease.org/EN/10.14336/AD.2018.0606

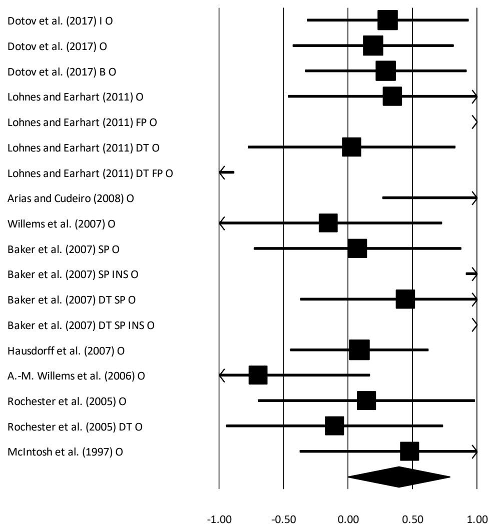

Supplemental Figure 13. Forest plot illustrating individual studies evaluating the effects of rhythmic auditory cueing on stride length among healthy old participants. A negative effect size indicated reduction in stride length; a positive effect size indicated enhancement in stride length. Weighted effect sizes; Hedge's g (boxes) and 95% C.I (whiskers) are presented, demonstrating repositioning errors for individual studies. The (Diamond) represents pooled effect sizes and 95% CI. A negative mean difference indicates a favorable outcome for control groups; a positive mean difference indicates a favorable outcome for experimental groups. (O: Old, Y: Young, FP: Fast paced, SP: Slow paced, DT: Dual-task, I: Isosynchronous, B: Biological variability, LG: Low groove, HG: High groove, INS: Instructions, Mt: Motivating feedback, NMt: Non-motivating feedback)

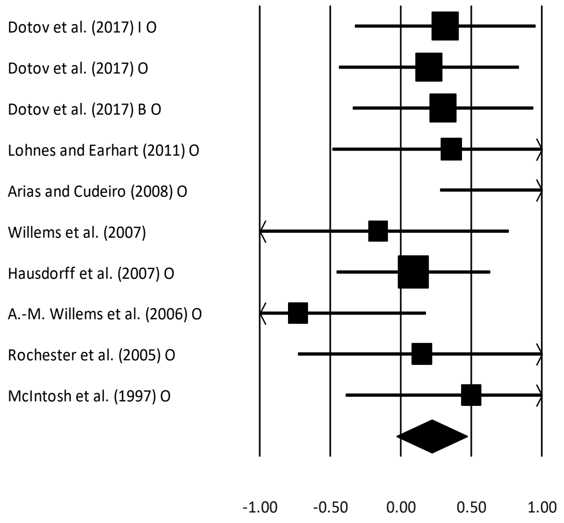

Supplemental Figure 14. Forest plot illustrating individual studies evaluating the effects of rhythmic auditory cueing on stride length among healthy old participants (non-modulated rhythmic auditory cueing). A negative effect size indicated reduction in stride length; a positive effect size indicated enhancement in stride length. Weighted effect sizes; Hedge's g (boxes) and 95% C.I (whiskers) are presented, demonstrating repositioning errors for individual studies. The (Diamond) represents pooled effect sizes and 95% CI. A negative mean difference indicates a favorable outcome for control groups; a positive mean difference indicates a favorable outcome for experimental groups. (O: Old, Y: Young, FP: Fast paced, SP: Slow paced, DT: Dual-task, I: Isosynchronous, B: Biological variability, LG: Low groove, HG: High groove, INS: Instructions, Mt: Motivating feedback, NMt: Non-motivating feedback)

© 2017. Chen W et al. Published online at http://www.aginganddisease.org/EN/10.14336/AD.2018.0606

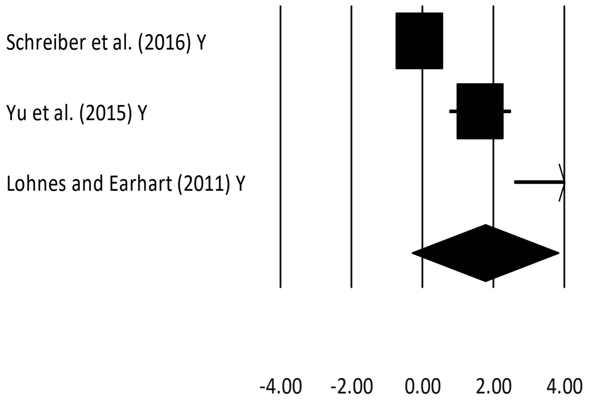

Supplemental Figure 15. Forest plot illustrating individual studies evaluating the effects of rhythmic auditory cueing on cadence among healthy young participants. A negative effect size indicated reduction in cadence; a positive effect size indicated enhancement in cadence. Weighted effect sizes; Hedge's g (boxes) and 95% C.I (whiskers) are presented, demonstrating repositioning errors for individual studies. The (Diamond) represents pooled effect sizes and 95% CI. A negative mean difference indicates a favorable outcome for control groups; a positive mean difference indicates a favorable outcome for experimental groups. (O: Old, Y: Young, FP: Fast paced, SP: Slow paced, DT: Dual-task, I: Isosynchronous, B: Biological variability, LG: Low groove, HG: High groove, INS: Instructions, Mt: Motivating feedback, NMt: Non-motivating feedback)

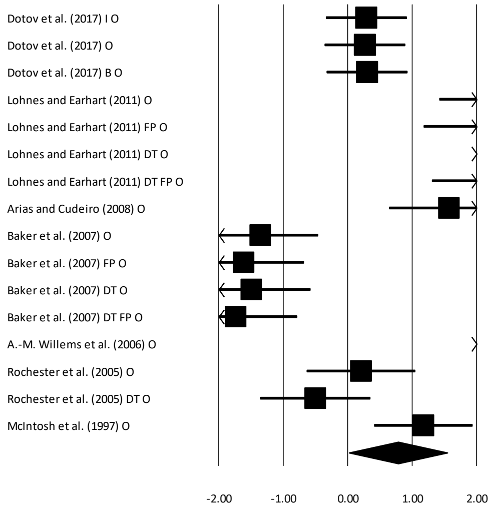

Supplemental Figure 16. Forest plot illustrating individual studies evaluating the effects of rhythmic auditory cueing on cadence among healthy old participants. A negative effect size indicated reduction in cadence; a positive effect size indicated enhancement in cadence. Weighted effect sizes; Hedge's g (boxes) and 95% C.I (whiskers) are presented, demonstrating repositioning errors for individual studies. The (Diamond) represents pooled effect sizes and 95% CI. A negative mean difference indicates a favorable outcome for control groups; a positive mean difference indicates a favorable outcome for experimental groups. (O: Old, Y: Young, FP: Fast paced, SP: Slow paced, DT: Dual-task, I: Isosynchronous, B: Biological variability, LG: Low groove, HG: High groove, INS: Instructions, Mt: Motivating feedback, NMt: Non-motivating feedback)

© 2017. Chen W et al. Published online at http://www.aginganddisease.org/EN/10.14336/AD.2018.0606

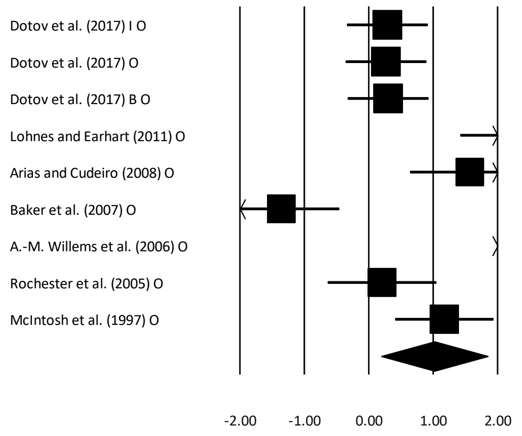

Supplemental Figure 17. Forest plot illustrating individual studies evaluating the effects of rhythmic auditory cueing on cadence among healthy old participants (un-modulated rhythmic auditory cueing). A negative effect size indicated reduction in cadence; a positive effect size indicated enhancement in cadence. Weighted effect sizes; Hedge's g (boxes) and 95% C.I (whiskers) are presented, demonstrating repositioning errors for individual studies. The (Diamond) represents pooled effect sizes and 95% CI. A negative mean difference indicates a favorable outcome for control groups; a positive mean difference indicates a favorable outcome for experimental groups. (O: Old, Y: Young, FP: Fast paced, SP: Slow paced, DT: Dual-task, I: Isosynchronous, B: Biological variability, LG: Low groove, HG: High groove, INS: Instructions, Mt: Motivating feedback, NMt: Non-motivating feedback)

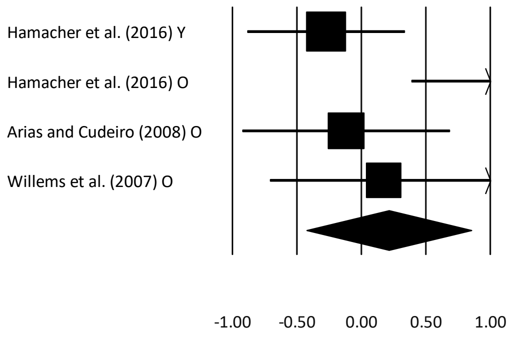

Supplemental Figure 18. Forest plot illustrating individual studies evaluating the effects of rhythmic auditory cueing on Coefficient of variability for stride time among healthy young & old participants. A negative effect size indicated reduction in Coefficient of variability for stride time; a positive effect size indicated enhancement in Coefficient of variability for stride time. Weighted effect sizes; Hedge's g (boxes) and 95% C.I (whiskers) are presented, demonstrating repositioning errors for individual studies. The (Diamond) represents pooled effect sizes and 95% CI. A negative mean difference indicates a favorable outcome for control groups; a positive mean difference indicates a favorable outcome for experimental groups. (O: Old, Y: Young, FP: Fast paced, SP: Slow paced, DT: Dual-task, I: Isosynchronous, B: Biological variability, LG: Low groove, HG: High groove, INS: Instructions, Mt: Motivating feedback, NMt: Non-motivating feedback)

© 2017. Chen W et al. Published online at http://www.aginganddisease.org/EN/10.14336/AD.2018.0606
